# Supplementary material for: The theoretical molecular weight of NaYF4:RE upconversion nanoparticles
Source: Sci Rep. 2018 Jan 18;8:1106. doi: 10.1038/s41598-018-19415-w (PMC5773537; doi:10.1038/s41598-018-19415-w)
Supplement: Supplementary file 1 — Supplementary information [file 41598_2018_19415_MOESM1_ESM.pdf]

Supplementary material:

The theoretical molecular weight of $NaYF_4$:RE upconversion nanoparticles

Lewis E. Mackenzie,[a] Jack A. Goode,[a] Alexandre Vakurov,[a] Padmaja P. Nampi,[b] Sikha Saha,[c] Gin Jose,[b] Paul A. Millner.[a]

[a] School of Biomedical Sciences, Faculty of Biological Sciences, University of Leeds, United Kingdom, LS2 9JT.

[b] School of Chemical and Process Engineering, Faculty of Engineering, University of Leeds, United Kingdom, LS2 9JT.

[c] Leeds Institute of Cardiovascular and Metabolic Medicine (LICAMM), Faculty of Medicine and Health, University of Leeds, United Kingdom, LS2 9JT.

Corresponding author: Lewis MacKenzie L.MacKenzie1@Leeds.ac.uk

Table S1: Standard atomic weight of elements typically utilised in $NaYF_4$:RE UCNPs. Data from Meija et al., (2013).[46]

Element	Standard atomic weight (AMU or g/mol)
(Na	22.989
F	18.998
Y	88.905
Yb	173.054
Er	167.259
Gd	157.25

Standalone Executable GUIs to calculate nanoparticle molecular weight

Two stand-alone GUIs were created to enable easy calculation of the theoretical molecular weight of UCNP (the simple GUI, see Figure S1), and the molecular weight of nanoparticles with arbitrary parameters (the advanced GUI, see Figure S2). These GUIs are shown in Figures S1 and S2. These GUIs are available from the University of Leeds Research Data Depository under a CC BY 4.0 licence and can be found and referenced via the following DOI: https://doi.org/10.5518/173.

These GUIs were developed as stand-alone applications for Windows PCs (Windows 7 or newer) and are not compatible with Macintosh or Linux PCs. The GUIs were compiled into stand-alone 64-bit executable files by using the MATLAB complier toolbox *deploytool* functionality. The GUIs were tested on 64-bit PCs running Windows 10. The first installation of a GUI was found to take around 45

minutes: this is primarily due to the automated download and installation of the required MATLAB runtime. After MATLAB runtime installation, the GUIs start-up time is typically < 10 seconds. Note that a MATLAB runtime compatible with MATLAB 2016a or newer is required to run these GUIs, and that MATLAB runtime versions older than 2016a may need to be removed from PCs prior to GUI installation.

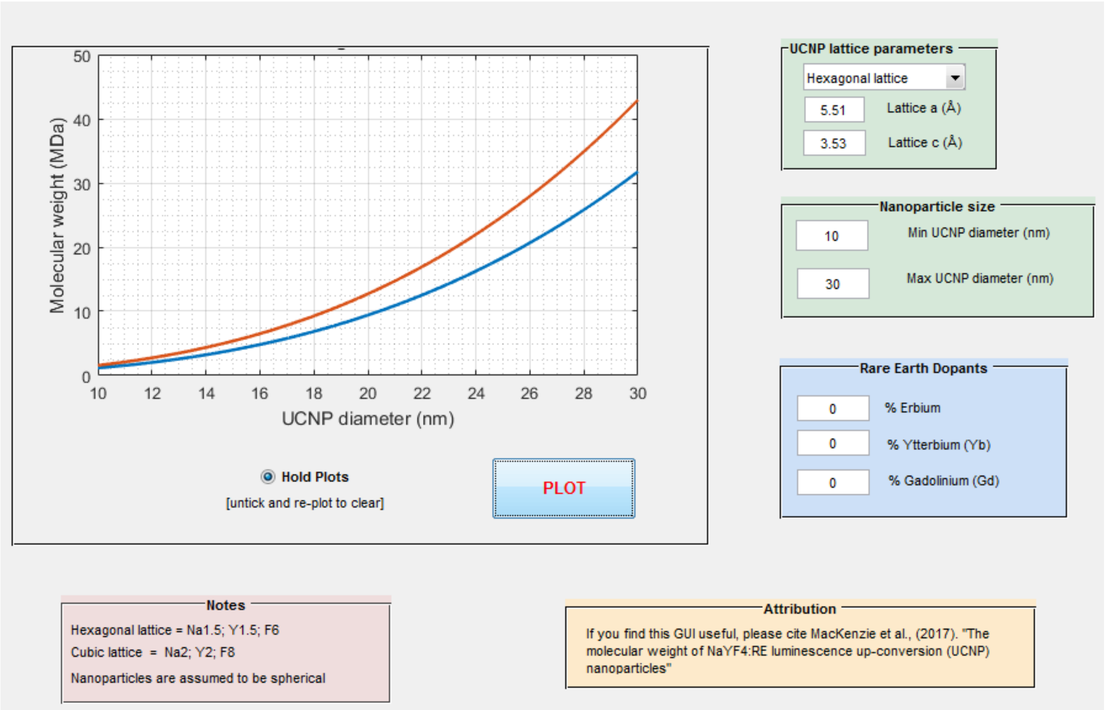

Figure S1. The basic GUI developed to estimate the molecular weight of NaYF4:RE UCNPs of spherical geometry and cubic or hexagonal lattice parameters.

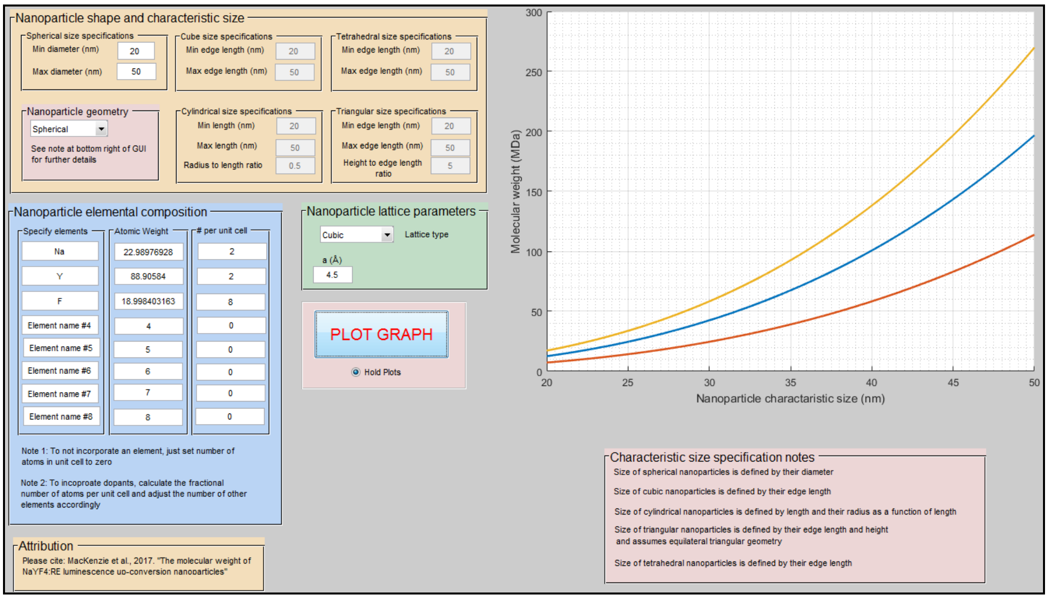

Figure S2. The advanced GUI developed to estimate the molecular weight of nanoparticles with arbitrary elemental constituents; arbitrary cubic or hexagonal lattice spacing; and arbitrary nanoparticle geometry.

Sedimentation Velocity Analytical Ultracentrifugation (svAUC) experiment

$NaYF_4$ UCNPs doped with 20% Yb and 2% Er were synthesized via the hydrothermal method as per the protocol in Zhang et al., (2014).[10] UCNP morphology was quantified by TEM imaging, and found to be approximately spherical with an average diameter of 32 ± 5 nm. XRD measurements revealed that UCNPs were cubic phase with a crystal lattice parameter a = 5.51 ± 0.01 Å. We estimate the theoretical molecular weight of these 32 nm diameter UCNPs to be ~ 43 MDa.

For svAUC measurements, UCNPs were suspended in standard 0.1M NaCl buffer at 3 concentrations: 0.71, 0.95, and 1.19 mg/mL, and placed within 2-sector aluminium cells assembled with sapphire windows. Using a Beckman XLI analytical ultracentrifuge the rotor chamber temperature was equilibrated to 20.0°C for 1 hour prior to data collection. Using a constant rotor speed of 3000 rpm, interference data was collected for thirty minutes, scanning at ~20 second intervals.

At all concentrations of UCNPs the concentration profile was observed to sediment with considerable back-diffusion. The commonly used Sedfit svAUC analysis program (Sedfit version 14.4d. Schuck et al., 2014. DOI: 10.1039/C3AN01507F), was not capable of fitting the experimental data. As an alternative analysis solution, custom Matlab algorithms were created to fit sigmoid line functions to the data in order to quantify the sedimentation and diffusion of UCNPs as the measurement progressed. The sedimentation coefficient for each sample was calculated by plotting radial position of the concentration boundary versus the product of angular velocity^2 (in radians) and time (in seconds). A line of best fit was plotted to this data by least squares fitting and the sedimentation coefficient was calculated as the gradient of this best fit line divided by 4π. The sedimentation coefficient vs. UCNP concentration was plotted for three samples and a best fit line plotted by least squares fitting. The zero-concentration sedimentation coefficient (S_0) was calculated as the y-intercept of this best fit line. However, S_0 was found to be negative, and thus could not be used for calculations of UCNP molecular weight using the theory described by Carney et al., (2011). Higher rotor speeds were tested to reduce back diffusion, however above 3,000 rpm material was pelleted to the bottom of the cell before data collection was possible.
